# The Humanistic Care Behaviour of Head Nurses and the Influence on Stress and Transition Shock of New Nurses: A Cross‐Sectional Study

**DOI:** 10.1002/nop2.70514

**Published:** 2026-04-10

**Authors:** Yuhong Luo, Bei Yun, Wenjing Ling, Wen Fang, Shoubi Wang, Chaoting Zhen, Yaling Li

**Affiliations:** ^1^ Department of Nursing Affiliated Hospital of Guizhou Medical University Guizhou China; ^2^ School of Nursing Capital Medical University Beijing China; ^3^ School of Nursing Fudan University Shanghai China

**Keywords:** humanistic care, job rotation, nurses, stress, transition shock

## Abstract

**Aim:**

To examine the interrelationships among head nurses' humanistic care behaviour, job rotation stress, and transition shock among new nurses in Southwest China.

**Design:**

This study employed a cross‐sectional observational research design.

**Methods:**

A total of 1128 new nurses were recruited from ten tertiary hospitals in Guizhou Province between March and September 2022. Participants were assessed using the Transition Shock of Newly Graduated Nurses Scale, Nursing Job Rotation Stress Scale, and the Head Nurses' Humanistic Care Behaviour Scale, and hierarchical regression analysis was used to identify the relationships.

**Results:**

Head nurses' humanistic care behaviour was negatively associated with transition shock (*r* = −0.417, *p* < 0.01), whereas job rotation stress was positively associated with transition shock (*r* = 0.731, *p* < 0.01). Stepwise regression and mediation analyses showed that head nurses' humanistic care behaviour and job rotation stress jointly explained 57.1% of the variance in new nurses' transition shock, with humanistic care behaviour reducing transition shock both directly and indirectly by alleviating rotation stress. These findings underscore the critical role of supportive leadership in easing new nurses' transition into clinical practice.

**Conclusions:**

To reduce new nurses' job rotation stress and transition shock, head nurses should receive structured training in humanistic care and incorporate empathetic leadership practices into daily management. Clinical strategies such as fostering emotional communication, offering timely support, and creating a feedback culture can facilitate smoother transitions and enhance nurse retention.

**Patient or Public Contribution:**

No patient or public contribution.

## 1 Introduction

A higher transition shock results in a higher turnover rate of new nurses, ultimately threatening patients' quality of care and safety. In Korea, the average turnover rate of new nurses is 29.0%, which is twice that of senior nurses (13.9%) (Korean Hospital Nurses Association 2015), while the turnover rate of new nurses in Taiwan in the first three months of employment has reached a staggering 54.8%–57.7% (Lee et al. 2009). The rising attrition rate of new nurses not only results in a waste of educational resources and increased employment costs but also creates significant challenges for hospital managers in recruitment, training, admission, and re‐recruitment, consequently increasing hospital management costs (Alanazi et al. 2023; Havaei et al. 2023). Meanwhile, owing to vacancies and the low efficiency of new nurses in their initial stages of work, the remaining nurses' work burden and pressure gradually increase, posing serious challenges to the stability and sustainability of patient care (Bae 2022; Kukkonen et al. 2020; Madolo and Hloba 2023).

Hospital administrators aim to improve nurses' capabilities by arranging job rotations (equivalent to cross‐training) to prepare registered nurses to deliver patient care in more than one clinical specialty at an institution (Huang et al. 2016). Job rotation is also expected to address nurse shortage issues arising from the difficulty in recruiting and retaining registered nurses and difficulties in hospital branch expansion in China (Lin et al. 2013). According to the “Chinese nursing development plan (2016–2020)” and “Training Syllabus for New Nurses,” new nurses are required to undergo a two‐year job rotation training.

As basic‐level managers, head nurses interact with new nurses more frequently, and their behaviours directly affect their work experiences (Kim and Yeo 2021; Yao et al. 2023). Head nurses with humanistic care ability are good at creating an open and fair organisational environment, guiding new nurses through instilling various psychological structures such as trust, positive emotions, and optimism, satisfying their basic psychological needs and effectively relieving pressure (Song 2018). Therefore, exploring the relationship between head nurses' humanistic care behaviour, nursing job rotation stress, and transition shock of new nurses is conducive to reducing the turnover rate and ensuring the stable development of nursing teams.

## 2 Background

The demanding clinical environment, complex interpersonal relationships, and the gap between theoretical knowledge and practical skills pose significant challenges for new nurses (Kreedi et al. 2022; Shi et al. 2023). This challenge encountered by new nurses during their transition from novice to professional nursing roles is termed transition shock. Duchscher (2009) developed an evaluation model for transition shock using the reality shock theory, encompassing physical, intellectual, emotional, developmental, and sociocultural dimensions. Labrague and De Los Santos (2020) revealed that the transition shock experienced by new nurses impacts both work outcomes (e.g., job satisfaction, stress, burnout, and turnover rate) and patient‐related outcomes (e.g., adverse nursing events and quality of care), significantly influencing nursing development. In addition, new nurses are important members of the nursing team. Therefore, identifying factors that influence transition shock is crucial to improving early retention and the quality of nursing practice.

Among these factors, job rotation stress has emerged as a key contributor. Nurse job rotation involves the transfer of nursing personnel among departments or units within the same hospital department without promotion or salary adjustments (Bolan et al. 2021; Chatterjee et al. 2023). According to the “Training Syllabus for New Nurses,” newly graduated nurses must undergo a two‐year job rotation training across four to five different departments before becoming registered nurses (i.e., internal medicine, surgery, intensive care unit, gynaecology, etc.) (Su et al. 2021). After the training, new nurses must pass an examination before officially becoming registered nurses. Job rotation training is important for new nurses to become qualified quickly. However, owing to frequent department rotations, heavy workloads, and frequent assessments, new nurses experience varying levels of pressure (Ren et al. 2021; Yao et al. 2023). Prolonged exposure to high pressure during the transition can lead to job burnout and even resignation (Alharbi et al. 2023; Pang et al. 2020).

The role of head nurses in this context warrants special attention. As front‐line managers, head nurses differ from mentors or clinical educators by holding administrative authority, being responsible for task assignment, workload balance, and emotional support (Brook et al. 2019). Their close proximity to both clinical operations and individual staff members places them in a unique position to influence new nurses' adjustment experiences (Brook et al. 2019). Unlike clinical preceptors who mainly guide technical skills, head nurses shape the organisational culture and climate through their management style (Gregg et al. 2023). Humanistic care behaviour, defined as the integration of empathy, respect, and individualised support into leadership practices, has been shown to foster a supportive work atmosphere, promote staff morale, and reduce turnover intentions (Song 2018; Gregg et al. 2023). Therefore, understanding the interrelationships among head nurses' humanistic care behaviour, job rotation stress, and transition shock is critical. Beyond confirming associations, this study also aims to explore the mediating role of job rotation stress in the relationship between leadership behaviours and transition outcomes. Based on the existing literature, this study hypothesises that head nurses' humanistic care behaviour is negatively associated with new nurses' transition shock, both directly and indirectly through the reduction of job rotation stress. Such insights may inform targeted interventions in nurse leadership training and strategies.

## 3 Methods

### 3.1 Design

This study used a quantitative, cross‐sectional observational survey design, following the STROBE checklist guidelines for reporting.

### 3.2 Participants

A quantitative, non‐experimental, cross‐sectional survey design with convenience sampling was adopted, recruiting new nurses from March to September 2022 at 10 tertiary hospitals in Guizhou Province. For the study's mediation framework using structural equation modelling (SEM), the sample size is calculated with the formula: *n* = 10 × *q* (Sousa and Rojjanasrirat 2011). Where *q* is the number of free parameters or observed variables. The model includes three latent variables (transition shock, job rotation stress, head nurses' humanistic care behaviour), 63 observed variables (scale items). Thus, *q* = 66, and the minimum sample size is: *n* = 10 × 66 = 660, To improve statistical power and account for dropout, a sample size of 200–400 is often recommended. For this study, a final sample size of 900–1200 participants are advised to ensure sufficient power and handle potential data issues. Inclusion criteria comprised new nurses who (a) passed the nurse qualification examination, (b) had a nursing licence, (c) had < 2 years of work experience, (d) received job rotation training, and (e) provided informed consent to voluntarily participate. Students were excluded from this study.

### 3.3 Instruments

#### 3.3.1 Demographic Questionnaire

The questionnaire encompassed age, sex, educational background (technical secondary school, junior college, bachelor's degree, or master's degree and above), marital status (married, single, or divorced), family location, working years (≤ 1 year, > 1 year), employment mode (formal establishment, contract system), professional title (no job title, nurse, or nurse practitioner), independent nursing work (yes or no), first major was nursing (yes or no), work experience in other hospitals (yes or no), and monthly income (< 3000RMB, 3000–6000RMB, 6000–9000RMB, and > 9000RMB).

#### 3.3.2 Transition Shock of Newly Graduated Nurses Scale

This scale, developed by Xue et al. (2015), evaluates new nurses' transition shock across physical (six items), psychological (eight items), knowledge and skills (five items), and sociocultural and developmental dimensions (eight items). These 27 items were scored on a 5‐point Likert scale (1 = completely disagree, 5 = completely agree), ranging from 27 to 135. The higher the score, the more serious the impact of transition shock. The reported Cronbach's *α* coefficient was 0.918, and the content validity was 0.906. And the scale was originally developed and validated in Chinese, and no further translation was required for this study.

#### 3.3.3 Nursing Job Rotation Stress Scale

This scale measures nurses' job rotation stress and was originally developed by a Taiwan scholar in 2016, It was culturally adapted to the mainland Chinese context in a 2020 study by Liu (2020). It consists of 10 items across the following three dimensions: emotions (four items), communication (three items), and daily life (three items). The items were scored on a 5‐point scale (1 = strongly disagree, 5 = strongly agree). The scores range from 10 to 50 points; the higher the score, the higher the nurses' job rotation stress. The reported Cronbach's *α* coefficient was 0.857.

#### 3.3.4 Head Nurses' Humanistic Care Behaviour Scale

This scale measures head nurses' humanistic care behaviour and was developed by Song (2018). This scale consists of 26 items across the following three dimensions: work support (13 items), interpersonal communication (eight items), and manager quality (five items). The items were scored on a 5‐point scale (1 = completely inconsistent, 5 = completely consistent). The scores ranged from 26 to 130; the higher the score, the greater the head nurses' humanistic care behaviour. The reported Cronbach's *α* coefficient was 0.908, and the content validity was 0.964. In this study, the instrument was completed by new nurses to reflect their perceptions of their head nurses' humanistic care behaviours, rather than being self‐reported by the head nurses themselves. Therefore, all results reflect nurses' perceived humanistic care behaviour. The scale was originally developed and validated in Chinese, and no further translation was required for this study.

### 3.4 Data Collection

The data for this study was collected from March to September 2022 in a hospital setting. After obtaining consent from the director of the hospital's nursing department, new nurses who met the inclusion criteria were selected. The survey was conducted using WENJUANXING (www.wjx.cn), and all questions were set as required answers. The questionnaire took about 15 min to complete. The research was conducted in a hospital setting, and the data collection was done by the designated groups responsible for each hospital. These groups attended an online collective meeting where they explained the inclusion and exclusion criteria for new nurses, the purpose of the investigation, informed consent, data confidentiality, and other related matters. Subsequently, the person in charge distributed the questionnaire to the new nurse training working group in the hospital and provided guidance to ensure new nurses filled out the questionnaire anonymously. In addition, the completion time was monitored, and questionnaires completed in an unusually short time were excluded. Incomplete responses and data with missing values were removed to ensure data quality. After all necessary checks, questionnaires from 1128 new nurses (94.00%) were included for statistical analysis.

### 3.5 Data Analysis

Data was analysed using SPSS 25.0 and SPSS AU software. Descriptive statistics, independent samples *t*‐test, and one‐way analysis of variance (ANOVA) were conducted using SPSS 25.0. Pearson's correlation was used to determine the direction and size of the relationships among transition shock, head nurses' humanistic care behaviour, and nursing job rotation stress. A multiple linear stepwise regression analysis was conducted using the total transition shock score of the transition shock of new nurses as the dependent variable. The independent variables included general demographic variables with statistical significance, head nurses' humanistic care behaviour, and nursing job rotation stress. Hierarchical regression analysis using SPSSAU explored the effect path of head nurses' humanistic care behaviour on new nurses' transition shock.

### 3.6 Validity and Reliability

All the questionnaires used in this study have been validated in previous research. The Cronbach's *α* coefficient of Transition Shock of Newly Graduated Nurses Scale was 0.918 with a content validity of 0.906. The Head Nurses' Humanistic Care Behaviour Scale demonstrated a Cronbach's *α* coefficient of 0.908, with a content validity of 0.964. The Nursing Job Rotation Stress Scale yielded a Cronbach's *α* of 0.857.

## 4 Ethical Considerations

In this study, confidentiality and anonymity were meticulously upheld. Initially, the researchers sought approval from the hospital's nursing department manager. Subsequently, nursing department assistants conveyed the study's purpose, methodology, and associated risks to potential participants via online management groups. Participants were encouraged to reach out to the researchers via email or telephone for any clarifications and were assured of their right to withdraw from the study at any point. All data were anonymised, coded, and processed. This study was approved by the Ethics Committee (REDACTED, Approval No. 2023 Lun Shen No. 966) and conducted in accordance with the principles outlined in the Declaration of Helsinki.

## 5 Results

### 5.1 Demographic Characteristics

This study obtained a sample of 1128/1200 new nurses, representing a participation rate of 94.0%. Among them, 72 respondents were excluded owing to incomplete responses. Most participants were under 25 years (755, 66.9%) and female (1006, 89.2%), with a small proportion of male participants (122, 10.8%). Most participants held a bachelor's degree (699, 62.0%), followed by a junior college education (415, 36.8%). Additionally, 772 (68.4%) had chosen nursing as their primary major at university, and 1025 (90.9%) had performed nursing work independently. Concerning monthly income, most participants (728, 64.5%) fell within the midlevel range in Southwest China.

The mean total transition shock score was 89.93 out of 135. Statistically significant differences were observed in transition shock concerning educational background (*p* < 0.01), sex (*p* < 0.05), and those with nursing as the first major (*p* < 0.01). However, no other demographic variables showed significant differences in transition shock (*p* > 0.05) (refer to Table 1).

**TABLE 1:** Comparison of the scores of new nurses' transition shock based on demographic characteristics (*n* = 1128).

Variables	Sample	Transition shock	*t/F*	*p*
*Age*				
≤ 25	755	89.19	2.057	0.125
> 25	373	91.43		
*Sex*				
Male	122	84.26	7.210	0.007
Female	1006	90.61		
*Educational background*				
Technical secondary school	4	72.25	3.299	0.020
Junior college	415	87.51		
Bachelor's degree	699	91.32		
Master's and above	10	100.00		
*Marital status*				
Married	981	90.27	1.199	0.302
Single	145	87.43		
Divorced	2	105.00		
*Family location*				
Local	541	88.45	3.713	0.054
Non‐local	587	91.29		
*Working years*				
≤ 1 year	782	89.93	3.142	0.896
> 1 year	346	89.26		
*Employment mode*				
Formal establishment	42	94.24	1.321	0.251
Contract system	1086	89.76		
*Professional title*				
No job title	168	89.51	1.532	0.205
Nurse	812	89.30		
Nurse practitioner	148	93.71		
*Independent nursing work*				
Yes	1025	89.76	0.357	0.550
No	103	91.32		
*First major is nursing*				
Yes	772	89.86	38.823	< 0.001
No	356	96.59		
*Work experience in other hospitals*				
Yes	456	88.22	3.657	0.056
No	672	91.09		
*Monthly income*				
< 3000RMB	249	92.33	1.034	0.376
3000–6000RMB	728	89.31		
6000–9000RMB	141	88.82		
> 9000RMB	10	91.30		

### 5.2 Head Nurses' Humanistic Care Behaviour, Nursing Job Rotation Stress, and Transition Shock

Table 2 illustrates the overall mean score of the Transition Shock of Newly Graduated Nurses' Scale. Moreover, the scoring rates for the dimensions of transition shock, ranging from high to low, were as follows: physical aspect, knowledge and skills, psychological aspect, and social culture and development. The overall mean score of the Nursing Job Rotation Stress Scale was 35.05 (SD = 0.70), with the communication and emotional dimensions exhibiting the highest and lowest score rates, respectively. The Head Nurses' Humanistic Care Behaviour Scale scored 98.58 (SD = 0.49), with the manager quality and work support dimensions recording the highest and lowest score rate, respectively. Head nurses' humanistic care behaviour and nursing job rotation stress significantly influence an individual's experience of transition shock. Head nurses' humanistic care behaviour displayed a significant negative correlation with new nurses' transition shock (*r* = −0.417, *p* < 0.01), indicating that an improvement in head nurses' humanistic care behaviour corresponded to a notable decrease in transition shock. Conversely, nursing job rotation stress exhibited a significant positive correlation with transition shock (*r* = 0.731, *p* < 0.01) indicating that an increase in nursing job rotation stress was associated with a corresponding increase in transition shock.

**TABLE 2:** The scores of head nurses' humanistic care behaviour and new nurses' nursing job rotation stress and transition shock (*N* = 1128).

Variables	Score range	Mean ± SD	%
New nurses' transition shock	27–135	89.93 ± 0.84	66.61
Physical aspect	6–30	21.88 ± 0.67	72.93
Psychological aspect	8–40	26.74 ± 0.72	66.85
Knowledge and skills aspect	5–25	16.85 ± 0.56	67.40
Sociocultural and development aspect	8–40	24.45 ± 0.74	61.13
Nursing job rotation stress	10–50	35.05 ± 0.70	70.10
Emotional	4–20	13.39 ± 0.71	66.95
Daily life	3–15	10.50 ± 0.41	70.00
Communication	3–15	11.17 ± 0.30	74.47
Head nurses' humanistic care behaviour	26–130	98.58 ± 0.49	75.83
Work support	13–65	48.21 ± 0.52	74.17
Interpersonal communication	8–40	30.70 ± 76.65	76.75
Manager quality	5–25	19.67 ± 78.68	78.68

### 5.3 Multiple Linear Stepwise Regression Analysis on Factors Influencing New Nurses' Transition Shock

Taking the head nurses' humanistic care behaviour and the nursing job rotation stress post as independent variables and the transition shock as dependent variables, the stepwise regression model was statistically significant and explained over half (57.1%) of the variance in new nurses' transition shock, highlighting the strong predictive power of head nurses' humanistic care behaviour and job rotation stress (Table 3).

**TABLE 3:** Multiple linear stepwise regression analysis on influencing factors of transition shock of junior nurses.

Independent variable	*B*	Standard error	Standard coefficient	*t*	*p*
Constant	50.818	5.204		9.766	< 0.01
Sex	1.228	1.564	0.015	0.786	0.432
Educational background	0.130	0.995	0.003	0.131	0.896
First major is nursing	1.698	1.098	0.032	1.546	0.122
Head nurses' humanistic care behaviour	−0.213	0.023	−0.198	−9.446	< 0.01
Nursing job rotation stress	1.577	0.050	31.752	31.752	< 0.01

### 5.4 Effect Path of Head Nurses' Humanistic Care Behaviour on New Nurses' Transition Shock

**FIGURE 1:**
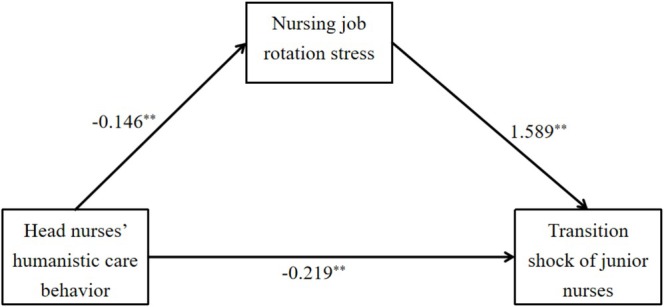
The mediating role of nursing job rotation stress. *p < 0.05, **p < 0.01.

Mediation analysis further revealed that job rotation stress partially mediated the relationship between head nurses' humanistic care behaviour and transition shock. This means that head nurses influence new nurses' adjustment not only through direct support but also indirectly by alleviating stress associated with job rotation (Table 4; see Figure 1 for the conceptual path model).

**TABLE 4:** Test of mediating effect model (*n* = 1128, *β*).

	*Y*	*M*	*Y*
*X*	−0.415**	−0.146**	−0.219**
*M*	—	—	1.589**
*R* ^2^	0.174	0.103	0.571
Δ*R* ^2^	0.173	0.103	0.571
*F*	*F* = 237.384, *p* = 0.000	*F* = 129.963, *p* = 0.000	*F* = 749.647, *p* = 0.000

*Note:* *X* = head nurses' humanistic care behaviour, *M* = nursing job rotation stress, *Y* = transition shock of junior nurses.

** *p* < 0.01.

## 6 Discussion

This study contributes to the literature by identifying both direct and indirect mechanisms through which head nurses' humanistic care behaviour influences transition shock, particularly within the context of nursing job rotation stress. Unlike prior studies that examined these variables independently, our research offers a more comprehensive understanding of the interplay between leadership, structural stressors, and nurse adaptation. Additionally, the focus on Southwest China provides insights into how institutional and regional contexts may shape the new nurse experience, a dimension often overlooked in existing research centered in economically developed areas.

### 6.1 Impact of Transition Shock on New Nurses

The findings revealed that the transition shock experienced by new nurses was at a moderately high level. The study sites, located in less‐developed regions of Southwest China, may partly explain this outcome (Su et al. 2021; Yun et al. 2023). Compared with previous studies conducted in more developed urban centers such as Shanghai and Wuhan, the transition shock levels observed in this study appeared relatively lower (Chen et al. 2017; Yao and Huang 2019). This discrepancy may be attributed to differences in institutional expectations—hospitals in less‐developed areas may place less emphasis on research output or professional advancement early in a nurse's career, thereby easing the adaptation process for newly graduated nurses.

Further analysis of the scores across all dimensions indicated that the physical aspect obtained the highest score. The physical aspect encompasses various physical changes, such as fatigue and sleep disturbances. New nurses often need to extend their working hours to fulfil clinical duties owing to unfamiliar working environments, unrefined clinical skills, and heavy workloads.

Consequently, frequent shifts can lead to sleep disruptions, back pain, and fatigue (Boni et al. 2022). Additionally, new nurses struggle to balance work, family, and social life, often sacrificing rest time to maintain relationships with family and friends (Su et al. 2021). Therefore, nursing managers should reasonably allocate workload and flexibly arrange working hours to assist new nurses in achieving work‐life balance and alleviate the physical aspects of transition shock.

In this study, knowledge and skill scores ranked second only to physical aspects. This outcome stems from the disjointedness between knowledge, skills, and reality, rendering new nurses unable to cope with complex clinical practice. Furthermore, the perplexity experienced by new nurses in nursing practice can manifest fear and stress due to insufficient specialised knowledge and skills (Chen et al. 2021). Consequently, nursing managers should assess new nurses' readiness for practice regarding professional knowledge and skills and offer personalised guidance in practice. Finally, the sociocultural and developmental aspects garnered the lowest scores. This trend may be attributed to the 68.4% of respondents who chose nursing as their primary choice, received sufficient support from society and family, had a higher sense of professional nursing identity, were more satisfied with the social status of nurses, and had a higher motivation and enthusiasm for work. Research indicates that transition shock obstructs new nurses' job adaptation, and the lack of professional identity contributes to a high turnover rate among new nurses (Cai 2021). Therefore, nursing managers should prioritise the sociocultural and developmental aspects of new nurses, aiding in enhancing their self‐efficacy and professional identity.

### 6.2 Impact of Nursing Job Rotation Stress on New Nurses

The nursing job rotation stress experienced by new nurses in this survey was at a high level. Within the Nursing Job Rotation Stress Scale, the dimension of communication emerged as the highest, indicating that communication accounted for the largest proportion of nursing job rotation stress among new nurses. Job rotation is viewed as a professional cross‐training strategy aimed at assisting nurses to expand their job scope, enhancing their work experience and skills, fostering professional development, and cultivating interpersonal relationships (Huang et al. 2016). However, for new nurses, rotating across multiple departments entails adapting to varied working modes, comprehending the requirements and regulations of different departments, and constantly acquainting themselves with new colleagues. Moreover, they must navigate relationships with patients, family members, and medical staff while also managing interactions with preceptors and head nurses, a task marked by anxiety, constant adaptation, and communication barriers. Therefore, we advocate reinforcing communication and coordination between department managers and staff to foster a harmonious department atmosphere. Additionally, leveraging the roles of mentors and peers can provide valuable support for new nurses.

### 6.3 Impact of Head Nurses' Humanistic Care Behaviour on New Nurses

The findings revealed that the score for head nurses' humanistic care behaviour among new nurses was lower than that in Song's survey of in‐service nurses (Song 2018), and the score rates for work support (74.2%), interpersonal communication (76.8%), and management quality (78.7%) did not reach higher levels. Notably, the scoring rate for work support was the lowest among the three dimensions. While this study did not directly assess head nurses' workload or institutional constraints, the lower perceived level of support may reflect structural challenges in clinical settings, such as high patient volume, staff shortages, and limited managerial availability. This underscores the importance for head nurses to recognise the significance of providing adequate support for the working environment of new nurses (Peng et al. 2022). Beyond focusing solely on nursing knowledge and skills, attention should also be directed toward addressing the humanistic care needs of new nurses during their transition process. Doing so can help mobilise their enthusiasm for work, mitigate turnover rates, and enhance the overall quality of nursing care.

### 6.4 Correlations Among Transition Shock, Nursing Job Rotation Stress, and Head Nurses' Humanistic Care Behaviour

The results indicated a significant correlation between new nurses' transition shock and nursing job rotation stress, with higher levels of nursing job rotation stress corresponding to elevated levels of transition shock. While nursing rotation aims to prepare new nurses for delivering patient care across various clinical specialties, prior research highlights the challenges nurses face during such rotations. Difficulties building interpersonal relationships, internal conflicts, feelings of helplessness, fear, frustration, and increased workload contribute to nursing job rotation stress (Henderson et al. 2013). Thus, nursing managers should adopt a scientific psychological decompression model, conduct early systematic psychological interventions for junior nurses, reasonably reduce stressors, and improve new nurses' stress resistance abilities to reduce the levels of transition shock.

Furthermore, head nurses' humanistic care behaviour was negatively correlated with the main influencing factor of the new nurses' transition shock, consistent with previous research results (Wenxia et al. 2021). Nursing managers can facilitate the successful transition of newly graduated nurses into the workplace by providing a supportive organisational environment (Cai 2021). Newly graduated nurses working in positive work environments have lower transition shock than those working in negative work environments (Cottle‐Quinn et al. 2022). Head nurses should create a supportive working environment by implementing humanistic management practices and paying attention to the humanistic caring behaviour of new nurses. Head nurses' humanistic care can be reflected in meeting new nurses' reasonable needs, guaranteeing their basic rights and interests, arranging working hours, strengthening technical training, respecting personalities, and correctly managing relationships between doctors and nurses. Ultimately, head nurses' humanistic care should encompass physical, psychological, and sociocultural support to help them manage their work‐life balance.

### 6.5 Direct and Indirect Effects of Head Nurses' Humanistic Care Behaviour on Transition Shock

This study confirmed a direct association between head nurses' humanistic care behaviour and new nurses' transition shock, as well as the mediating role of nursing job rotation stress in this relationship. Humanistic care behaviour emerged as a key protective factor, helping new nurses manage both stresses associated with job rotation and the broader challenges of professional transition. Given the pressures new nurses face during this critical period, head nurses play a pivotal role in shaping a supportive work environment. By incorporating humanistic care into daily management practices, they can enhance psychological safety, promote job satisfaction, and reduce transition shock. These outcomes may be facilitated through targeted interventions, such as onboarding programs that include humanistic leadership training, structured peer mentoring, and stress monitoring mechanisms. At the organisational level, integrating humanistic care principles into nurse manager training curricula and performance evaluation systems may contribute to long‐term improvements in nurse retention and workforce resilience.

### 6.6 Limitations

This study has some limitations. First, the use of convenience sampling may limit the generalisation of the findings. To enhance the robustness and applicability of future research, it is recommended to adopt more rigorous sampling methods and include participants from diverse geographic and institutional contexts. Second, while this study affirmed the protective role of head nurses' humanistic care behaviour against new nurses' transition shock and nursing job rotation stress, future investigations must explore the impact of humanistic care behaviours exhibited by other hospital staff members (e.g., clinical teachers, preceptors, senior nurses, and doctors) on new nurses. In addition, individual factors and organisational variables (clinical settings, workloads, and head nurses' leadership style, etc.) should also be considered. Third, as data collected through self‐reported questionnaires, there is a potential risk of response bias, including the halo effect. Participants' perceptions may have been influenced by social desirability or subjective impressions, which could affect the accuracy of the responses. Lastly, the cross‐sectional design restricts our ability to infer causality between variables. Longitudinal or experimental studies are needed to examine how these relationships evolve over time and to establish clearer cause‐and‐effect pathways.

## 7 Conclusion/Implications for Practice

This study examined the influence of head nurses' humanistic care behaviours on new nurses' job rotation stress and transition shock. The findings confirmed that humanistic care directly reduces transition shock and indirectly alleviates it by easing job rotation stress. These results underscore the importance of cultivating a supportive and empathetic work environment led by humanistic nurse leaders. Practical recommendations include improving workplace infrastructure, ensuring transparent resource and performance management, enhancing emotional communication, and implementing structured feedback systems with senior staff. Additionally, integrating humanistic care training into leadership development programs can enhance job satisfaction and retention among new nurses, ultimately promoting a more stable and resilient nursing workforce.

## Author Contributions

Yuhong Luo and Yaling Li: Study design. Yuhong Luo and Bei Yun: Data collection and analysis and manuscript drafting. Wenjing Ling, Wen Fang, Shoubi Wang and Chaoting Zhen: Revision of the manuscript for important intellectual content.

## Funding

This study was supported by the Special Research Project for the Nursing Discipline at Guizhou Medical University (Grant No. YJ22027) and the Guiyang Science and Technology Planning Project ([2022]‐4‐2‐2).

## Conflicts of Interest

The authors declare no conflicts of interest.

## Supporting information


**Data S1:** STROBE Statement—Checklist of items that should be included in reports of *cross‐sectional studies*.

## Data Availability

The data that support the findings of this study are available from the corresponding author upon reasonable request.
